# Teaching home safety skills to children with autism spectrum disorders

**DOI:** 10.3389/fpsyt.2025.1688922

**Published:** 2025-11-28

**Authors:** Ayşe Mortaş Kum, Hatice Bilmez

**Affiliations:** European University of Lefke, Mersin, Türkiye

**Keywords:** autism, father training, preschool education, safety skills, video modeling

## Abstract

Children diagnosed with autism spectrum disorder (ASD) are 2 to 3 times more likely to experience injury or abuse than their typically developing peers of the same age. Parents have limited knowledge about the home safety skills of children with autism and often do not know how to respond in risky situations. In addition, fathers are generally less involved in the educational processes of children with ASD compared to mothers. The purpose of this study was to evaluate the effectiveness of the video modelling presented by fathers in teaching home safety skills (avoiding chemicals) to children with ASD. This study designed a single-subject multiple probe design with probe phases across four preschool-aged children with ASD (ages 3–4) and their fathers. Results demonstrated that all children acquired, generalized, and maintained the targeted safety skill with 100% success after six instructional sessions. Social validity findings indicated positive perceptions from fathers.

## Introduction

The World Health Organization (1) stated that accidents usually occur in the child’s natural environment, in or around the home. Burns, falls, and poisoning are the most common types of accidents. For these reasons, it is seen that accidents that may occur in the home are among the most important risk-bearing health problems worldwide. When the literature is examined, it is stated that home accidents constitute an average of 18-25% of all accidents, and approximately 45.4% occur among preschool children (2). Thus, both typically developing children and children with Autism Spectrum Disorder (ASD) are at fatal risk due to accidents that may occur voluntarily or involuntarily (2–4). It is reported that children diagnosed with ASD have difficulties in self-expression, making eye contact, initiating and maintaining conversation, interacting with peers, and pointing to objects, as well as having limitations in verbal behaviors (2). This increases the risk of daily home accidents for children with ASD (5). It is seen that children with ASD are more vulnerable to domestic accidents and are more exposed to the dangers that may occur (6, 7). Thus, children with ASD have difficulties in acquiring safety skills and demonstrating a learned skill due to their behavioral characteristics (8). Because of these challenges, teaching safety skills to children with ASD is vital for preventing potential injuries and ensuring their independence in daily life.

It is important to inform the parents of preschool children with autism and to provide them with the necessary training. Considering the characteristics given above, parents have a great role in the acquisition of safety skills in the preschool period. It is also important to teach safety skills to preschool children with ASD so that they can react appropriately and protect themselves against accidents that may occur in the community or at home (9, 10). To prevent or eliminate these risks, parents should have the necessary awareness and take preventive measures to prevent accidents that may occur (11). In addition to these measures, it is important to teach safety skills, as preschool children with ASD have difficulties recognizing safe or unsafe danger zones, asking for help, and most importantly, how to escape from these zones (2, 12–15). It is known that parents have insufficient knowledge about accident prevention and protection in terms of domestic safety skills and that young children with autism have a higher risk of encountering accidents (6, 13).

It is seen that parents have problems about what kind of safety measures to take when faced with home accidents (16). To prevent these dangers or to gain safety skills, one or more effective methods are used (17). There are also studies (e.g., 16, 18–21) that tested the effectiveness of the methods and techniques used in teaching safety skills to adults and teenagers with ASD. These are reinforcement systems, prompts, role playing, social stories, using technology and feedback on performance (22). In addition to these methods, another effective method in teaching safety skills is the ‘video modeling’ (23). Video modeling is a teaching method in which a behavior or a skill that is targeted to be taught is exhibited through an adult or a peer model, and the action exhibited is recorded on the video recording, the child watches these video recordings and puts the behaviors they watch into action (13, 24, 25).

Research on safety skills (5, 16, 20, 26, 27) shows that safety skills are vitally important for children and adults with developmental disabilities. Potter (28), who investigated father-child communication and interaction in children with ASD, concluded that fathers’ behavior towards their children with ASD, appreciation of their children, and the father’s role have a significant impact on the child’s personal development. When the studies conducted in Turkey are examined, it is seen that the studies conducted with father-children with ASD, especially the studies conducted using the video modeling method, positively affect father-child communication and interaction (Diner 14, 29–31).

In teaching abduction trap in teaching safety skills to children with ASD (24), to respond appropriately to chemical substance avoidance (32), in teaching the skill of crossing the street using an overpass (33), in teaching evacuation safety (25), firearms evasion (13), There are studies in which video modeling model is effective. Thus, considering the research on the video model teaching method, more research should be done on the prevention and acquisition of security threats, considering that it is an effective method in gaining security skills. The effectiveness of the video model teaching method in teaching children with ASD daily life skills such as social skills, language, communication, and safety (e.g. 24) revealed that the video model teaching method is effective in teaching abduction skills to children with ASD. When the studies conducted in Turkey were examined, it was revealed that the studies examining child-father communication and interaction (Diner 29, 31), especially the studies conducted using the video model method, were positive.

When the literature is examined, it is seen that while there are studies testing the effectiveness of the methods and techniques used in teaching safety skills to adults with ASD (e.g., 16, 18, 20, 21), there is a limited number of studies on preschool children with ASD (27). While there are limited studies (e.g., 16, 21, 27) on teaching safety skills to adults with intellectual disabilities, there are no studies in which the parents of preschool children with ASD, especially the fathers, are actively involved in the practice. It was found that there are limited studies in the world and no studies in Turkey in which the effectiveness of the video modeling method presented by fathers in teaching safety skills to preschool children with ASD was tested. In the Turkish Republic of Northern Cyprus, there is no study examining the effectiveness of the video model teaching method presented by fathers in teaching domestic safety skills to children with ASD. This study, which investigates the effectiveness of the video model teaching method presented by fathers in teaching home safety skills to children with Autism Spectrum Disorder, is thought to contribute to the field by filling the gap in the literature. Within the scope of the research, answers to the following questions were sought:

- Is the video modeling method presented by fathers effective for preschool children with ASD to learn home safety skills (staying away from chemical substances)?
- Is the video modeling method presented by fathers effective on children’s maintenance of home safety skills (staying away from chemical substances) one, three, and five weeks after the instruction?
- Is the video modeling method presented by the fathers effective on children’s use of the home safety skills (staying away from chemical substances) taught to them in different environments, people, and tools?
- What are the father participants’ views on the social validity of the study?

## Method

### Participants

Three boys and a girl with ASD and their fathers participated in this study. Family interviews showed that the participants were diagnosed in North Cyprus health institutions. Begining of the study, the Turkish version of the Gilliam Autism Rating Scale-2 (GARS-2-TR), developed by Gilliam in 1995 and adapted and standardized for use in Turkey by Diken, Ardıç, and Diken (34), was applied based on information given by fathers, and autism scores of the participants were calculated. All participants received 4 hours of individual special education in a special education school and attended a preschool on weekdays with neurotypical children.

To participate in this study, the prerequisite characteristics of the participants were determined as (a) being diagnosed with autism, (b) visual perception, (c) being able to follow verbal instructions, (d) being able to direct attention to visual and auditory stimuli for 5 minutes, (e) being able to direct attention to images on the computer/tablet screen for 3 minutes, (f) being able to imitate gross and fine motor skills.

Child1 is 3 years old. A GARS-2-TR test showed that his autism score was 93. Child1 is at par with his peers in terms of gross and fine motor skills. He responds to his name, makes eye contact, and can follow two-step verbal instructions. He has no words. Child1 has difficulties with social communication and safety skills. He focuses his attention on an activity for 15 minutes.

Child2 is 3 years old. A GARS-2-TR test showed that his autism score is 86. He makes eye contact, responds to his name, and follows two-step verbal instructions. He has no words. Child2 has gross and fine motor skills similar to his peers. He has difficulties with social communication and safety skills, and he focuses his attention on an activity for 15 minutes.

Child3 is 4 years old. A GARS-2-TR test showed that his autism score was 88. She has gross and fine motor skills similar to her peers. She responds to her name, makes eye contact, and can follow one-step verbal instructions. She has no words. Child3 has difficulties with social communication and safety skills. She focuses her attention on an activity for 15 minutes.

Child4 is 4 years old. A GARS-2-TR test showed that his autism score was 92. He makes eye contact, responds to his name, and can follow two-step verbal instructions, but is not sufficiently able to communicate or respond to questions. Child4 has difficulties with social communication and safety skills. He has gross and fine motor skills comparable to his peers, and he focuses his attention on an activity for 12 minutes.

The prerequisite characteristics of the fathers were (a) having a child diagnosed with ASD between the ages of 3-6, (b) being able to comprehend the auditory and visual information prepared for safety skills, (c) volunteering for the study, and (d) being able to use technology effectively. The four fathers who met the prerequisite and volunteered to participate in the study were aged between 34 and 39 years. Two fathers are high school graduates, and the other two hold a bachelor’s degree. One of the fathers is a police officer, and the others work freelance.

The research was conducted by the researcher, working as a lecturer in the Child Development Department of a university in North Cyprus. A postgraduate student from the field of special education collected the reliability data for this study.

### Settings and materials

In the first stage of the study, the researcher conducted one-on-one training sessions on video modeling and safety skills with the fathers in a seminar room in the university unit. After the first stage of the study, the researcher collected probe data from children in the individual training room of the Research and Education Centre for Children with Special Needs (ÖÇEM) unit affiliated with the state university. Afterwards, fathers held video modeling training sessions with their children at the individual training room of the ÖÇEM. There was a student table and two small chairs for children and a material cabinet. The classroom floor was covered with rubber. Generalization and follow-up sessions were carried out in the same individual training room at ÖÇEM.

During the father training, PowerPoint presentation slides introducing the video modeling method and safety skills, a laptop for the presentation of the slides, and a digital video camera were used for the preparation and watching of video images. The baseline, probe, generalization, and follow-up data collection form and the video modeling method instruction planning form were used to record the data obtained by the fathers regarding the performance of their children. In the introduction phase, which is the first phase of the father training in the study, there are plates, forks, bread, tomatoes, cucumber, cheese, and napkins for the ‘sandwich’ preparation skill. Liquid soap, a towel, and a sink were included for the ‘hand washing’ skill. For the ‘spreading chocolate on bread’ skill, a plate, a knife, chocolate, and bread were included. Finally, an empty Domestos (a kind of thick bleach) bottle, a microfiber cloth, a table, and a chair were used in the ‘staying away from chemicals’ skill in the instructional sessions. Additionally, in case the participant children did not have expressive language skills, the reporting behavior, which was part of the dependent variable of the study, was carried out using an alternative communication tool, which is a red colored card with a size of 7x7 cm with a big black cross. All respective materials were also used in the baseline, instructional, probe, generalization, and follow-up sessions.

### Research design

This study aims to reveal the effect of the video modeling method presented by fathers in teaching home safety skills (staying away from chemical substances) to children with autism spectrum disorder. This research was carried out with the multiple probe model with a probe phase between participants, one of the single-subject research models. Multiple probe models are a research model that allows the evaluation of the effectiveness of a teaching/behavior change intervention in multiple situations and, unlike multiple baseline models, it is not necessary to collect continuous baseline data (35, 36). In multiple probe models across behaviors, experimental control is established by ensuring that the change in the first behavior targeted to be taught occurs only with the application of the independent variable, that no change is observed in other behaviors to which the independent variable is not applied, and that the effect in question is repeated diachronically in other behaviors targeted to be taught in the study (35, 36). The interviews conducted with fathers to collect social validity data were evaluated subjectively.

### Dependent and independent variables

The dependent variable of this study is ‘Avoiding chemicals’, which is one of the home safety skills. Avoiding chemicals is defined as not touching the chemical substance, staying away from the environment where the chemical substance is present, and reporting the situation to an adult. In the related skill set, there are three types of possible responses in probe, generalization and follow-up sessions: (a) correct response (Moving away from the chemical within two minutes without touching the bottle of chemical on the table and report to his/her father that there was a dangerous substance on the table (children who do not have expressive language skills should give the danger card on the table to their father), (b) incorrect response (If the child didn’t do even one of the following behaviors: not touching the chemical substance, moving away from the environment, reporting to an adult, the child was marked as an incorrect response on the data collection form. For example, touching the bottle of chemical substance on the table was considered an incorrect response and the child’s response was interrupted. The session ended. (c) no response (If the child didn’t respond within two minutes, it was considered an incorrect response). On the other hand, the independent variable of this study is the ‘Video Modeling Method presented by the fathers’.

### Experimental procedure

The experiment of this study began after the fathers’ training on preparing videos, and they had acquired the skills to provide video modeling. The training given to fathers was provided by the researcher. Baseline sessions, full probe sessions, and follow-up sessions were conducted by the researcher. Generalization sessions were conducted by a student who had a master’s degree in special education. Instructional sessions were run by the fathers.

### Training fathers

The researcher trained fathers to teach their children chemical avoidance, a home safety skill, through video modeling. Fathers’ training was presented in three stages as (a) learning to prepare video images, (b) learning to apply the video model teaching method, and (c) learning safety skills.

The first two stages of the training process were related to the Video Modeling Method. In the first stage, the researchers taught the fathers how to create video images. In the second stage, the fathers were taught how to implement the video modeling method. In the third and last stage, they were informed about what a safety skill is and its classifications.

The training on learning to prepare video given to the fathers was carried out through (a) introduction, (b) modelling, and (c) providing feedback. The training on practicing the video modeling method was carried out through (a) introduction, (b) modelling, (c) role playing and providing feedback, that is, behavioral skills teaching, while the training on safety skills consisted only of the introduction phase. At the end of the training, the video that would be used in the experimental process of the research was prepared by the father. The video was prepared for the skill of avoiding chemicals. Adult models were used in the video.

In the scenario in the video, while a young man is eating snacks at the table, an adult man comes and wipes the table. Then, this man leaves the bottle of chemical he used on the table and walks away from the environment. The young man who is eating his snacks sees the bottle of chemical on the table, he picks up the danger sign card on the table without touching the bottle. The young man takes this card to adult man and gives it to him. Then, the young man takes the adult man by the hand and brings him to the area where the chemical is. The adult man picks up the bottle of chemical from the table and removes it from the area.

### Baseline probe sessions

Before starting the instructional sessions of the study, a probe session was organized, and baseline data were collected. The sessions were conducted at least five consecutive times until stable data were obtained. The correct and incorrect responses of the child were recorded by putting a ‘+’ sign for correct responses and a ‘-’sign for incorrect responses in the child’s responses column in the probe sessions data collection form. The researcher conducted the baseline probe sessions in the individual education room in the ÖÇEM. The researcher created a reason to go to the education room (e.g., ‘Let’s eat these nice snacks in the classroom.’) and then the researcher and the child sat at the table together. After seating the child at the table, the researcher gave him/her a snack. While the child was eating the snack, the researcher pretended to wipe the table and put the empty chemical bottle (filled with water) on the table and left the room. The researcher was not in the environment where the child was present, but was positioned in such a way that she could see whether the child touched the chemical substance. When the child responded correctly, the child was reinforced, and when the child responded incorrectly (e.g., touched the chemical bottle), the researcher immediately came to the environment, ended the session, and removed the chemical bottle. This process was carried out in the same way with all participants.

### Instructional sessions

These instructional sessions were organized as one session per day, 3 days a week. The sessions were conducted by fathers in an individual training room at the ÖÇEM. During the sessions, the setting was organized with a table where the father and the child could comfortably watch the video and two chairs where they could sit side by side. Before the father brought his child to the room, he made the computer ready to watch the video. Afterwards, he brought his child to watch the video. The father provided his child with an attention-getting cue (e.g., ‘Now we are going to watch the video with you. Are you ready?’) and then, if he heard a sign or verbal expression from his child that he was ready (e.g., nodding his head, saying ‘I am ready’), he reinforced his child verbally (e.g., ‘Well done’, ‘You are great’).

The father started the video and then checked whether the child’s attention was on the screen. After watching the video, the father put the empty container of the chemical substance on the table by putting snacks on the table for the child to have a snack, and pretended to wipe the table. Then the father moved away from the table and started to watch the child. Then, the child was expected not to touch the chemical substance on the table within two minutes, to move away, to take the danger card and report it to the father, and to bring the adult to the room where the chemical substance was present. The correct responses of the child were verbally reinforced (e.g., ‘Good for you, you are super, you are great’) and the chemical bottle was taken from the table by the father and put in the cupboard. Where the child reacted incorrectly in this process, the father stopped the child from performing the skill and ended the session by saying (‘Well, this is enough for today, we will continue again tomorrow’). This session continued until the child received 5 full points three times in a row in the target skill in the probe sessions. The researcher took the necessary notes about the implementation carried out by the fathers and, feedback was provided to the fathers after each implementation session. The reason for this was that any wrong implementation during the practice of safety skills could lead to ethical violations, so it was decided to give this feedback.

### Full probe sessions

Where the first participant performed at an eligible level with the criterion, full probe sessions were organized respectively. In the full probe sessions, data were collected simultaneously from each child participant. The full probe sessions were conducted by the researcher in a similar way to the baseline sessions. In the full probe sessions, data were collected until at least five sessions of stable data were obtained. After obtaining stable data in the probe sessions, before working with the second child, father training sessions were organized for the second father, and then the instruction sessions were started. After the criteria were met in the second participant child, the same process was repeated with the third and fourth participants.

### Follow-up and generalization sessions

After the criterion was met in the instructional sessions, follow-up sessions were organized after 1, 3, and 5 weeks to determine to what extent the participants maintained the targeted skills. The data related to the follow-up sessions were collected by considering the process followed in the probe sessions. The follow-up sessions were conducted in the homes of the participant pairs. The sessions were conducted like the baseline sessions. To determine the generalization levels of the participant children’s ‘avoiding chemicals’ safety skill, which was taught by using the video modeling method presented by the fathers, to different people and different tools, generalization sessions were held as a pre-test-post-test. Generalization pre-test-post-test sessions were conducted simultaneously with each participant child by the researcher in an individual training room in the ÖÇEM where the baseline sessions were also conducted. After five sessions of stable data were obtained in the baseline sessions, pretest generalization data were collected before starting the application. Post-test generalization data were generated upon the finalization of the instructional session and when the criteria were met. The follow-up and generalization sessions were conducted in a similar way to the baseline sessions.

### Data collection

#### Effectiveness data

Effectiveness data were analyzed by examining the correct and incorrect responses of the child participants. Three types of data were collected in the study: effectiveness, reliability (inter-observer and treatment reliability data), and social validity data.

Data were collected to determine the effectiveness of the video modeling method presented by the fathers on the participant children’s skill to avoid chemicals. The researcher used a scoring system to evaluate the child’s performance. A scoring chart was created out of 5 for the dependent variable. It was accepted as a criterion that the participants scored at least 4 points three times in a row. This scoring was adapted from Lumley et al. (37). ‘Not touching the chemical substance, moving away from the environment, reporting to an adult, bringing the adult to the environment where the chemical substance was found -5 points’, ‘Not touching the chemical substance, moving away from the environment, reporting to an adult but not bringing the adult to the environment where the chemical substance was found -4 points’, ‘Not touching the chemical substance, moving away from the environment but not reporting to an adult and not bringing the adult to the environment -3 points’, ‘Not touching the chemical substance, taking the x mark in hand but not reporting to the adult and not moving away from the environment -2 points’, ‘Not touching the chemical substance but continuing to stay in the environment and not reporting to the adult -1 point, and finally’ Taking the chemical substance in hand and not interacting with it -0 points. Data were collected through direct observation during the implementation sessions, while video recordings were used for reliability checks. The collected data were graphed as daily probe data in the instruction phase.

The data collected by the researcher regarding the skills exhibited by the children were plotted on the line graph. Moreover, follow-up data were collected to show the accuracy level of the children after one, three, and five weeks, after the fathers of the participant children acquired the safety skills taught by applying the video modelling teaching method.

#### Inter-observer reliability and treatment reliability data

At least 30% of the baseline, probe, generalization, and follow-up sessions were determined unbiasedly for each participant to determine the inter-observer reliability for the dependent variable of ‘avoiding chemicals’ for the participant children. The dependent variable for the children participants was the skill of ‘avoiding chemicals’; thus, to determine the treatment reliability data, the researcher analyzed and evaluated the video recordings to identify the inter-observer reliability data for all children within the scope of the study. Additionally, treatment reliability data were evaluated by filling in the form ‘+’ for the steps carried out by the researcher and ‘-’for the steps that could not be carried out by the researcher.

### Social validity data

The social validity data collection form was comprised of questions indicating the function and importance of the target skill aimed to be taught to the father participant. Moreover, the questions for the determination and revelation of the sustainability and effectiveness of the process of teaching the target skill are fourteen in total. Eleven of these questions consist of closed-ended questions such as ‘Yes, Partially Yes, Neutral, Partially No, and No’. The last three of the closed-ended questions have a ‘Yes, No’ question style. Therefore, in case the fathers answered ‘Yes’ to the last 3 questions, they were asked an open-ended question related to this question (‘What kind of problems did you face?’).

### Data analysis

The findings obtained at the end of the research were analyzed through graphical analysis. In the graph where the research findings were analyzed, the horizontal axis shows the time dimension and represents the number of sessions organized in the research. The vertical axis is the quantitative expression of the dependent variable, and in this research, the quantitative expression of the dependent variable is expressed as the score received by the subjects out of 5 points on the vertical axis.

The data collected for the participating children were determined by examining the calculation of ‘Agreement/Agreement + Disagreement X 100’ in the analysis of inter-observer reliability data (38). The inter-observer reliability coefficient for the participating children was calculated as 100% in all sessions.

Treatment reliability data for the sessions of the video modeling method presented to the children through their fathers were determined using the formula ‘Observed Father Behavior/Preplanned Father Behavior X 100’ (39).

A percentage of non-overlapping data analysis was conducted for the effect size calculated between the two phases regarding the effectiveness of the video modeling instruction presented by the fathers. According to the percentage of non-overlapping data analyzed in the study, the percentage of non-overlapping data for Child1 was 88.8%, while the percentage of non-overlapping data for Child2, Child3, and Child4 was 100%.

Consequently, all sessions conducted by the researcher were 100% reliable in all participant children. Finally, the social validity data collected to determine the fathers’ opinions about the purpose of the research, the training planned for this purpose, the importance of the research, the teaching method, and the results were analyzed through descriptive analysis.

## Results

**Figure 1:**
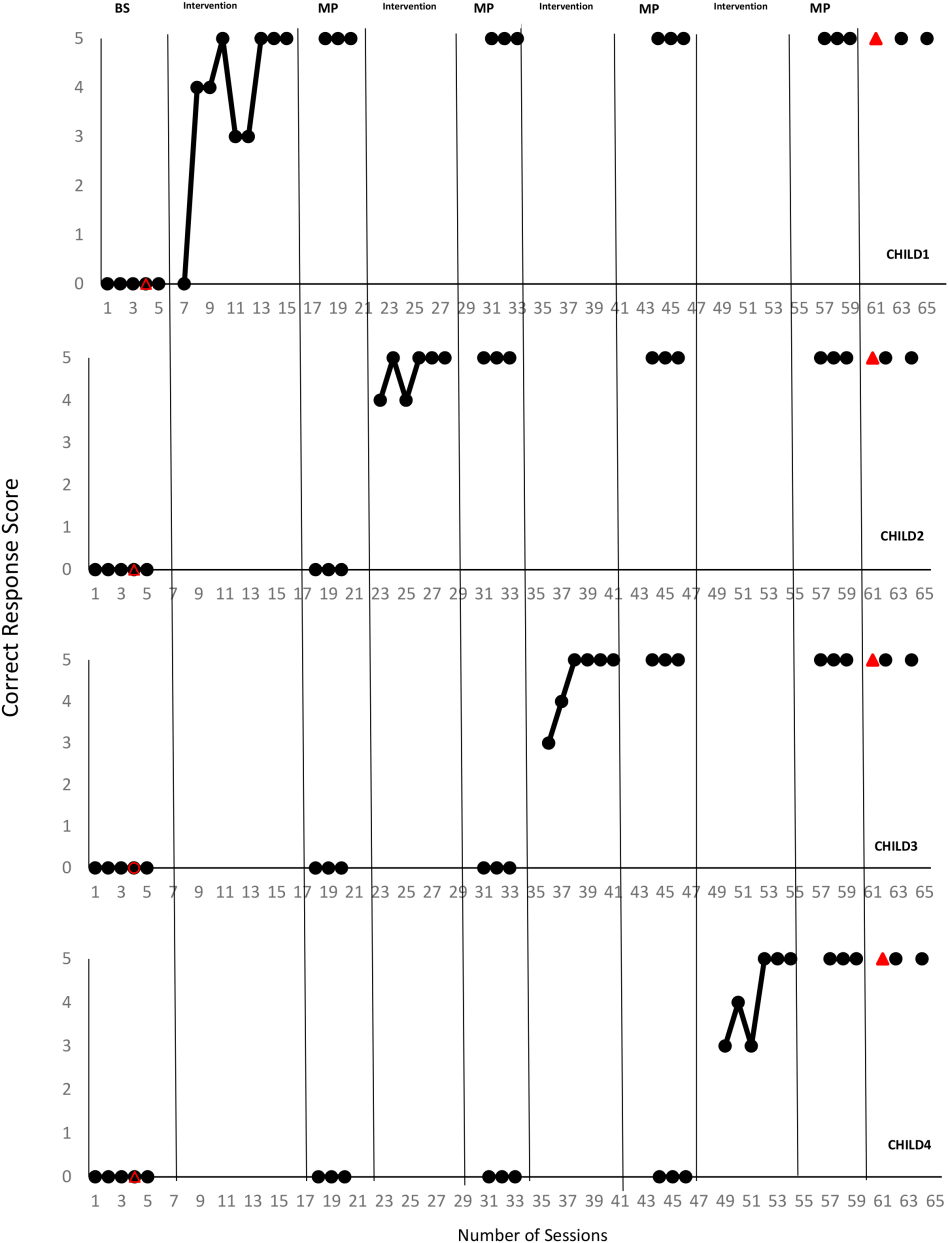
The results of the effectiveness of video modelled instruction.

The ‘baseline sessions, probe, generalization, and follow-up sessions’ of the children participants were examined, and the findings regarding the effectiveness of the video modeling method presented by the fathers in teaching the skill of ‘avoiding chemicals’, one of the targeted home safety skills, were evaluated respectively. The findings related to the instruction, generalization, and follow-up data of Child1, Child2, Child3, and Child4 “avoiding chemicals”, are presented in **Figure 1**.

Hence, Child1 did not exhibit a correct response (0-point level) for the skill of ‘avoiding chemicals’ in the baseline sessions. Pursuant to the analysis of Child1’ s instruction session, he learnt the skill at an average level (at the level of 3.77 points) as a result of nine instruction sessions. After meeting the criterion for the skill targeted to be taught by 5 points. Child1 was able to maintain it (at the level of 5 points) in the follow-up sessions held in the instruction sessions organized one, three, and five weeks later. Where Child1’s pre-test-post-test generalization data between different tools, and different people are examined, it is concluded that he generalized the skill (at the level of 5 points) (See **Figure 1**).

Child2 could not perform (at the level of 0 point) the skill of ‘avoiding chemicals’ in the baseline sessions. Where Child2’s instruction session was analyzed, he exhibited the skill at an average level (at the level of 4.67 points) following six instruction sessions. He was able to maintain the targeted skill (at the level of 5 points) in the follow-up sessions conducted in the re-organized instruction sessions after one, three, and five weeks, upon meeting the targeted skill at a rate of 5 points. As the pre-test-post-test generalization data of Child2 between different tools and different people are investigated, he generalized the skill at the level of 5 points (See **Figure 1**).

Child3 could not perform (at the level of 1 point) the skill of ‘avoiding chemicals’ in the baseline sessions. In terms of the instructional session, she performed the skill at an average level (4.80 points) as a result of six instructional sessions. She was able to maintain the targeted skill (at the level of 5 points) in the follow-up sessions conducted in the re-organized teaching sessions after one, three, and five weeks, upon meeting the targeted skill at a rate of 5 points. When Child3’s pre-test-post-test generalization data between different tools and different people are examined, it is seen that she generalized the skill (at the 5-point level) (See **Figure 1**).

Child4 was unable to exhibit the skill of ‘avoiding chemicals’, which is the target behavior at the baseline sessions. Considering the analysis of Child4’s instructional sessions, he performed the skill at an average level (4.50 points) following six instructional sessions. He was able to maintain the targeted skill (at the level of 5 points) in the follow-up sessions conducted in the re-organized instruction sessions after one, three, and five weeks, as he met the targeted skill at a rate of 5 points. The pre-test-post-test generalization data of Child4 between different tools and different people conclude that he generalized the skill (at the 5-point level) (See **Figure 1**).

In consideration of the father participants’ answers, they all expressed their positive opinions on the questions specified in the social validity form.

**Figure 2:**
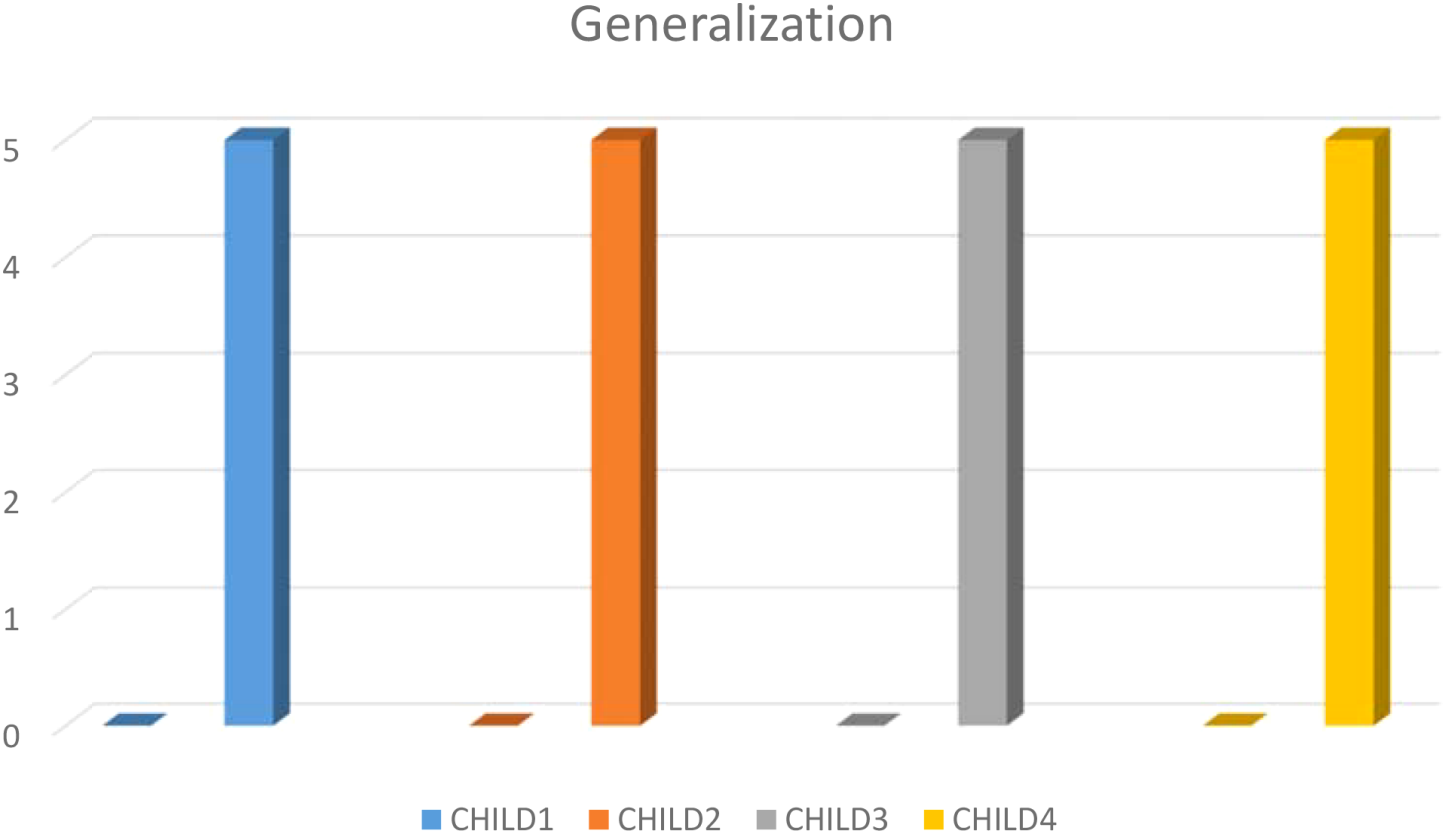
Pre-test and post-test generalization results obtained from childern.

When the pre-test generalization data were examined, it is seen that all participant children received ‘0’ points but post-test generalization results showed that all participant children with ASD received ‘5’ points and learned safety skills %100 (**Figure 2**).

## Discussion

This study examined the effectiveness of the video modeling method presented by fathers in teaching home safety skills to preschool children with autism. Additionally, social validity data were also evaluated by examining the opinions of the participant fathers who completed the research process regarding the effectiveness of this program. According to the social validity findings about the fathers, the use of the video modeling method facilitated positive results regarding the teaching of domestic safety skills to their children at the end of the research. The findings from the father participants show that they all learnt to use video modeling in teaching home safety skills to their children and were able to maintain it. The study findings are consistent with the findings obtained from studies in the literature on the effectiveness of video modeling for children with autism (24, 29, 40–44; Şafak, 2017; 45–47).

There are many studies on the effectiveness of the video modeling method for children with autism. In consideration with the literature, there are studies where video modeling was effective in teaching play skills (40, 41), imitation skills (29), protection from strangers (44), table wiping skills (47), preventing abduction to children with autism (24). Our study findings are consistent with such literature findings.

Bandura’s (48) social learning theory confirms that role modeling, reinforcement, observational learning, imitation, and prompting methods maximize individuals’ learning levels. This study supports the effectiveness of video modeling in helping children with autism acquire safety skills through observation.

Where the literature on safety skills is examined, Miltenberger, Sanchez & Valbuena (49) emphasized active learning approaches as effective strategies in their research, and thus different approaches to evaluation and training give effective results. Moreover, Miltenberger, Sanchez & Valbuena, (49) identified that studies on abduction, sexual abuse, poisoning, and firearm injuries, which are included in the classification of safety skills, yielded effective results. This research is expected to enhance the literature on teaching home safety skills.

The study by Tavukçu (50) concluded that positive results were achieved in teaching the target safety skill by using the steps of the behavioral skills teaching with the coaching offered to the mothers of children with autism. Tavukçu (50) also studied behavioral skills teaching practice with mothers of children with autism and obtained positive results in safety skills. Similarly, in our study, teaching safety skills to children with autism was presented by fathers and positive results were obtained, respectively, which is considered a contribution to the literature. In Şentepe & Kahriman’s (12) study, the mother participants showed high levels of positive results in the diagnostic scale scores of the safety measures taken when domestic accidents were examined. In this study, the skill of ‘avoiding chemicals’, one of the home safety skills, was examined, and positive results were revealed, which is in parallel with the research findings.

Where the findings of the research conducted with fathers are analyzed, Evren & Karabulut (51) identified that father-delivered video modeling instruction was effective on the level of imitation of object actions of students with autism. In this study, similar results were obtained with the result that the video modeling method presented by fathers to children with autism was effective. Similarly, our study noted that the video modeling method presented by fathers to children with autism is effective in teaching home safety skills.

The study by Şölen (52) concluded that the effectiveness and perception of the father training program, in which fathers with pre-school children of 3–6 years old are included in the process, on lifelong learning and family attitudes were examined and that fathers are enthusiastic about lifelong learning and there is a positive increase in the democratic attitudes of father participants. Moreover, the overlapping part of Şölen (52) and our study is that it is effective in children’s learning with the involvement of fathers in the education of preschool children. According to Rudelli, Straccia & Petitpierre (53), when the roles of fathers in the development of their children with autism were examined, there was a positive increase in the relationship between their perceptions and their feelings about their parenting experiences (self-efficacy, caring power, and satisfaction). As per the results, fathers have a positive effect on their young children with autism. The overlapping aspect of this study is that it is effective in working with fathers of children with autism.

Bordini et al. (54) used video modeling to teach joint attention and eye contact skills to children with intellectual disabilities and autism, and found that it positively impacted their social communication skills. They also found that video modeling, along with providing children with repetition and visual support, increased their learning level and motivation. Similarly, our study found that providing children with visual support through video modeling increased children’s motivation and had a positive impact on the acquisition of safety skills.

Research findings by Komen and Onginjo (55) revealed that fathers play a crucial role in children’s development and education, positively enhancing children’s development and motivation. Similarly, our study observed that children with autism were highly motivated when learning safety skills under the guidance of their fathers.

Zhong’s (56) study, which included fathers, concluded that the communication, interaction, and social bonds fathers establish with their children are positive, and that fathers’ role modeling increases children’s social interactions. Our study, however, reveals that fathers teaching their children safety skills as role models allows children with autism to generalize safe behaviors more easily.

The study results show that the father participants were able to reliably prepare and apply the images of the video recording with 100% accuracy. In this direction, the father participants’ treatment reliability revealed a high level of teaching. The study concluded that the father participants were effective in achieving a high level of implementation reliability in the participant children’s learning of the target skill. The literature shows that the studies (44, 45, 50, 52, 53, 57–61) on the presentation and effectiveness of teaching through the video modeling method offered by mothers and fathers are effective and reveal positive results. Thus, when the research findings are examined, the video model teaching method can be taught to parents, and it supports the results of the literature that families can reliably apply the video modeling method to their children with autism.

On the other hand, there are also examples of studies where scientifically proven practices such as simultaneous prompting and video modeling (62), incidental teaching, and teaching practices with discrete trials (63) were taught to mothers and fathers. Nonetheless, no research has evaluated the effectiveness of the video modeling method used by fathers to teach children with autism essential home safety skills.

Looking at the international literature on safety skills (24, 58), teaching processes with different age groups and different methods (53, 57) were included, but no research like this study was identified accordingly. Thus, this study is considered original, which would shed light on new research. In consideration with the findings of this study, the safety skill, which was aimed to be taught with the video modeling method presented by the fathers in the study, was applied in the same number of instruction sessions and as a result of 6 sessions in total, all participants exhibited the skill of staying away from chemical substances, one of the domestic safety skills targeted to be acquired, with 100% accuracy.

This study examined the effectiveness of a father-delivered video modeling intervention in teaching safety skills to preschool children with ASD. To generalize the findings, further research could examine the effectiveness of parent-delivered video modeling intervention in teaching different skills to children with different disabilities and demographic characteristics. Furthermore, studies could be conducted comparing the effectiveness and efficiency of mother and father delivered video modeling interventions.

Although the findings of this study on treatment reliability, effectiveness, and social validity were positive, there were also several limitations. First, the effectiveness of the training program on video modeling, offered to the fathers, was not tested using experimental control. This is a limitation of this study. In the future, researchers should use experimental controls to identify more effective and efficient ways of teaching the video modeling method to parents of children with autism. Moreover, future research can be planned to look in to whether fathers can generalize their video modeling teaching skills to other home safety situations or settings. Future research can examine the effectiveness of video modeling provided by family members in teaching more complex safety skills.

## Data Availability

The raw data supporting the conclusions of this article will be made available by the authors, without undue reservation.
